# Connexin's Connection in Breast Cancer Growth and Progression

**DOI:** 10.1155/2016/9025905

**Published:** 2016-08-23

**Authors:** Debarshi Banerjee

**Affiliations:** Department of Pediatrics, Columbia University Medical Center, Columbia University, 1130 St Nicholas Avenue, New York, NY 10032, USA

## Abstract

Gap junctions are cell-to-cell junctions that are located in the basolateral surface of two adjoining cells. A gap junction channel is composed of a family of proteins called connexins. Gap junction channels maintain intercellular communication between two cells through the exchange of ions, small metabolites, and electrical signals. Gap junction channels or connexins are widespread in terms of their expression and function in maintaining the development, differentiation, and homeostasis of vertebrate tissues. Gap junction connexins play a major role in maintaining intercellular communication among different cell types of normal mammary gland for proper development and homeostasis. Connexins have also been implicated in the pathogenesis of breast cancer. Differential expression pattern of connexins and their gap junction dependent or independent functions provide pivotal cross talk of breast tumor cells with the surrounding stromal cell in the microenvironment. Substantial research from the last 20 years has accumulated ample evidences that allow us a better understanding of the roles that connexins play in the tumorigenesis of primary breast tumor and its metastatic progression. This review will summarize the knowledge about the connexins and gap junction activities in breast cancer highlighting the differential expression and functional dynamics of connexins in the pathogenesis of the disease.

## 1. Introduction

Gap junctions are intercellular membrane channels that maintain direct intercellular communication through the exchange of ions, small molecules, and cellular metabolites between neighboring cells. Gap junction channels are formed at the basolateral surfaces of two cells with separation gap of 2-3 nm and connect directly to their cytoplasm [1]. One gap junction channel is composed of two hemichannels or a connexon. Each connexon, in turn, is formed through the hexameric oligomerization of proteins called connexins. Connexins are the multigene family of transmembrane proteins and they are the structural unit of gap junctions. So far, 21 connexin isoforms have been identified in humans [2, 3]. Each of these connexin isoforms constitutes four hydrophobic transmembrane helices, two extracellular loops (EL-1 and EL-2), a cytoplasmic loop (CL), and a carboxyl terminal (CT) and an amino terminal (AT) end; both termini are located at the cytoplasmic side [4, 5]. All of the connexin isoforms show highly conserved sequence similarities within the four transmembrane domains, two extracellular loops, and amino terminal (AT) end. Therefore, a highest degree of sequence diversity is seen mainly in the sequence and length of carboxyl terminal (CT) ends and cytoplasmic loops (CL). The extracellular EL-1 and EL-2 are the most conserved residues and they are required for proper docking interaction of the hemichannels from two adjacent cells for the channel (gap junction) to form [4, 6].

To date, there are 21 connexin genes in the human and 20 connexin genes in the mouse (Table 1) have been identified [7, 8]. Among these connexin genes, nineteen have similar orthologs in both the mouse and human genome [8]. There are some connexin genes that are only present in the mouse (Cx33) or in the human genome (CX25 and CX59) [7, 8]. Human genome contains two connexin pseudogenes that are related to the genes for GJA1 (CX43) [7] and CX31.9 [7, 8]. But mouse genome has not been detected for the presence of connexin pseudogene so far. Both human and mouse Cx23 are present in the respective genome and predicted from database [7, 8]. However, the gene has not been detected in transcriptional and translational level so far. The general gene structures of connexins are simple. There are two exons, exon 1 and exon 2, which are separated by an intron of variable size. Exon 1 contains 5′-untranslated region (5′-UTR) and exon 2 contains complete protein coding sequence and the 3′-untranslated region (3′-UTR) [7–9]. However there are several connexin genes that follow more complex genomic structure. Currently, there are two nomenclatures for connexin [7, 8]. In one nomenclature, connexins are named according to their molecular weight (MW). They are abbreviated as “Cx” followed by a suffix that indicates the approximate molecular weight of the protein in kilo daltons (kDa). For example, the Cx43 is a connexin protein that has the molecular weight of 43 kDa. Different connexins with similar molecular masses are denoted with a decimal point to distinguish them, for example, Cx30 versus Cx30.3 and Cx31 versus Cx31.1. In the second nomenclature, connexins, based on their sequence similarity and length of cytoplasmic domain, are divided into subgroup *α*, *β*, or *γ* [7, 8]. Furthermore, connexins are abbreviated as “Gj” for gap junction and serially numbered according to the order of their discovery.

Connexons (hemichannels), from adjacent cells, can interact with each other via several ways [9]. A hemichannel can be homomeric (single connexin isoform) or heteromeric (multiple connexin isoforms). Two identical homomeric channels can interact and form homotypic channels and when two different homomeric hemichannels interact to form a gap junction channel, it is known as a heterotypic channel. Connexins are expressed in almost all tissues with the exception of red blood cells, some neurons, and spermatozoa. In vertebrates, many tissues express two or more connexins. For example, vertebrate heart expresses Cx40, Cx43, and Cx45 [10–12]. Some connexins are very specific and some connexins are very abundant in terms of their tissue specific expression. For example, Cx43 is one of the most abundant connexins in the body as more than 35 tissues have been reported to express this protein [10, 12].

## 2. Biophysical and Biochemical Properties of Connexins

Biophysical and biochemical properties of connexins are regulated by several factors such as permeability of channel, voltage and chemical gating, and posttranslational modification of connexin proteins. Gap junctions are aqueous channels that are permeable to several small ions including Ca^2+^, small metabolites such as ATP, ADP, IP3, sugars, and small proteins with molecular weight less than 1 KDa [1, 3]. The permeability properties of connexin channels differ and depend on the connexin isoforms that compose the channel [13–15]. The permeability of a channel also depends on the amino acid residues and segments that line the pore [16]. Numerous studies with biological and nonbiological tracer molecules of various size, mass, charge, and properties have been able to reveal significant information about pore properties of different gap junction channels. Some of these studies suggested the following ranking of pore diameter: Cx43 > Cx32 > Cx26 > Cx37 > Cx46 [13]. Some connexins (Cx40 and Cx43) channels are selective to cations [14, 17] whereas some channels (Cx32) prefer anions to pass through them [14]. Gap junctions composed of Cx43, Cx40, and Cx45 show similar selectivity to monovalent cations K^+^ and Na^+^ [14, 17, 18].

The gap junction channels are also regulated by voltage gating and chemical gating. Gating of a channel is often used to refer to opening or closing of a channel. The connexin gap junction channels are sensitive to voltage fluctuation [19]. Junctional channels are sensitive to (i) inside-out or transmembrane voltage (*V*_*i*-*o*_ or *V*_*m*_) and (ii) transjunctional voltage (*V*_*j*_). Unitary conductance of connexin channels depends on connexin isotype and ranges from 14 ps to 300 ps [3, 20]. Connexin43 unapposed hemichannels have been shown to open at potentials greater than 60 mV with conductance of 220 ps [21]. pH or chemical changes (chemical gating) in the cell or microenvironment also influence opening or closing of a channel. Intracellular acidification has been shown to uncouple Cx26 [22], Cx32 [23], Cx38 [24], Cx43 [25], Cx46 [26], and Cx62 [27] gap junction channel. However pH gating of a channel also depends on connexins isoforms that compose the channel. For example, most channels composed of Cx43 and Cx46 are actively open at pH 7.2 [25, 26] whereas Cx62 channels are mostly closed at the same pH [27]. Gap junctions are also sensitive to Ca^2+^ gating. Increase in the intercellular Ca^2+^ has been shown to uncouple gap junctions in number of tissues to regulate their physiological properties [14, 28]. Exogenous chemicals and agents can also lead to chemical gating to exert their pharmacological effects in the intracellular milieu.

Posttranslational modification of connexins plays a major role in the regulation of biochemical properties of gap junction channels. Chemical modification of connexins such as phosphorylation, ubiquitination, acetylation, hydroxylation, S-nitrosylation, and palmitoylation has linked gap junctions to several physiological processes of a tissue [14]. Phosphorylation is the most well studied posttranslational modification of connexin proteins [29]. Cx43 has been documented to have 21 putative phosphorylation sites [14]. They are primarily serine residues but some tyrosine and threonine residues have also been identified as phosphorylation residues [14, 30, 31]. Several kinases including protein [kinase A (PKA), protein kinase C (PKC), p34cdc2/cyclin B kinase, casein kinase I (CK1), mitogen-activated protein kinase (MAPK), and pp60src kinase (src)] have been documented to cause phosphorylation of Cx43 [14, 30–32]. Phosphorylation or dephosphorylation at specific sites of Cx43 protein has been shown to be involved in electrical and metabolic coupling of gap junction channels. For example, ischemic preconditioning of isolated rat hearts led to a 34% decrease in maximal rate of uncoupling which was accompanied with a diminished total Cx43 dephosphorylation [14]. Phosphorylation of Cx43 by the protein kinase C enzyme leads to channel closure and a decrease in GJIC in lens epithelial cells [33]. Connexin can also act independently of its gap junction activity. A plethora of studies for the last several years have shown that connexins can modulate a cell's activity by interacting with key mediators of signaling pathways such as cytoskeletal proteins, enzymes, and signaling messengers [34, 35]. There is increasing evidence that gap junctions or connexins function as a signaling complex to regulate function and transformation of a single cell or group of neighboring cells in the environment [36].

Connexins have also been implicated in the pathogenesis of cancer. Dysfunctions of connexins have been linked with several adult cancers such as melanoma [37], skin cancer [38], pancreatic tumor [39], prostate cancer [40], lung tumor [41], and breast tumor [36, 42]. The current review will focus on the summarizing the knowledge of connexin expression and function in the normal mammary gland development and in tumorigenesis of breast tumor.

## 3. Connexins in Mammary Gland Development

Human mammary gland is an intricate organ that is composed of glandular, fatty, and fibrous tissues. Mature mammary gland consists of a series of alveoli that are organized into milk producing glands called lobules [42]. Each lobule is connected towards the nipple via ducts that transport milk from the lobules to the nipple. A single layer of luminal epithelial cells surrounds the ducts and alveoli and a basal myoepithelial cell layer surrounds the epithelium at the surface. A layer of fatty tissue surrounds the breast glands and extends throughout the breast. The major development and differentiation of the mammary glands occur postpuberty [42, 43]. During involution and pregnancy, the mammary gland also undergoes extensive differentiation and remodeling to attain a lactating structure. From birth to postpregnancy, the development of mammary glands is regulated by the signal pathways that include hormones, local growth factors, and interactions between epithelial cells with surrounding stroma [43, 44].

The gap junctional intercellular communication (GJIC) also plays a major role in the proper development, differentiation, and functioning of vertebrate mammary glands (Table 2) at different stages of growth, from postpuberty to postpregnancy. Human mammary glands have been shown to express two connexin isoforms, Cx43 and Cx26 [45, 46]. In addition to these connexins, mouse mammary glands have been found to express two more connexin isoforms, Cx30 and Cx32 [47, 48]. The expression, localization, and channel formation of all these connexins are distinct and controlled in a precise manner throughout the mammary gland development. Cx26 is the first connexin identified in human mammary glands. Several studies have shown that Cx26 channels are predominantly located in between the luminal cells indicating its selective function in luminal cell proliferation [49].

Another connexin, Cx43, is found to form gap junction channels only between myoepithelial cells and is speculated to maintain myoepithelial differentiation in resting human mammary gland [49]. In the mouse, two other connexins, Cx30 and Cx32, are also expressed in the luminal cells during lactation [47, 50]. The expression of Cx30 and Cx32 is not detected in human mammary glands which indicates that these connexins may have a distinct function in mouse mammary glands that is either compensated or not required in human counterparts.

## 4. Functions of Connexins in Mammary Gland

The mammary gland gap junction channels formed by different connexins have distinct functions. Many studies have shown that connexins expression in the rodent mammary gland modulates during pregnancy, lactation, and involution. Cx26 and Cx32, which are spatially distributed at the basolateral borders of luminal cells, were detected at all developmental stages of mammary gland [36, 42]. Cx26 and Cx32 have increased expression, at the mRNA and protein levels, during lactation and then declined in involution. At early stages of mammary gland development, Cx26 functions in luminal epithelial cell proliferation and, in later stages of development, along with Cx32, Cx26 is required for the proper production of milk by secretory cells [36, 49, 51]. Cx30, which is selectively expressed in epithelial cells, showed a peak expression at the onset of lactation in mice [51]. Cx30 was found to be colocalized with Cx26; however its expression decreases with concomitant increase of Cx32 expression at parturition suggesting physiological importance of differential connexins expression at the specific developmental stages of the mammary gland [36, 49, 51]. Cx43 was found to localize to myoepithelial cells and it is required for proper functioning of this cell type during lactation [49]. Cx43 expression is decreased during mid-pregnancy and lactation; however phosphorylated [52] Cx43 is active during lactation. A study conducted by El-Sabban et al. 2003 [52] shows CID-9 cells, grown under differentiating conditions, exhibited a decrease in Cx43 mRNA expression but concomitant increase in protein levels suggesting Cx43 is posttranslationally regulated during mammary gland development and differentiation.

Mammary gland connexins, Cx26 and Cx32, can compensate for each other's function [42, 51]. Conditional knockout of Cx26, during pregnancy, does not affect the normal mammary gland function [42]. Similarly, Cx32 null mice are associated with proper mammary gland development and functioning [42, 51]. Gap junction channels can be formed by heterohexamer containing Cx26 and Cx32, in the luminal epithelial cells, during the later stage of pregnancy with stoichiometric ratio greater for Cx26 [42]. During lactation the ratio of Cx32 increases within the hexamer and eventually homomeric Cx32 is formed [42]. The change of Cx26-Cx32 connexon to Cx32 connexon is speculated to be in accordance with the cell's biological need. Channels formed by Cx32 alone are much wider than the heteromeric Cx32-Cx26 channels and allow the free passage of larger molecules such as cAMP and cGMP [53, 54], the metabolites that act in the several pathways involved in the regulation of mammary gland growth and differentiation. The function of Cx30 has not been investigated to date and elucidation of the function of Cx43 is impaired due to the fact that Cx43 knockout is lethal in mouse embryos [55]. However some studies with knock-in technology shed light on the role of Cx43 in breast development. Heterozygous Cx43KICx32 mice (where an allele of Cx43 gene is replaced with Cx32 allele) show normal milk production but impaired milk ejection indicating a possible role of Cx43 in the functioning of the mammary gland [56].

## 5. Connexins as Breast Tumor Suppressors

Cx26 and Cx43 are well documented for their tumor-suppressive roles in several carcinomas, including breast tumors. Cx26 and Cx43 have been deemed breast tumor suppressors since 1991 when Lee et al. [45] first identified them as the candidates for tumor suppressor genes by subtractive hybridization techniques. However, till today, the correlation of expression of connexins with the function as a tumor suppressor at different stages of breast carcinogenesis is far from clearly understood and is often contradictory. Nonetheless, the current notion, which is supported by ample research evidences, identifies connexins as tumor suppressors in breast cancer pathogenesis (Table 2). Connexins had been found to be downregulated or poorly expressed in human breast cancer cell lines, such as MCF-7 and MDA-MB-231, in both protein and mRNA levels [36, 42]. Studies in the early 90s detected low levels of Cx26 and Cx43 mRNA in the primary cells derived from human breast tumors and several breast cancer cell lines [57, 58]. Human or mice breast tumors also exhibited reduced connexins expression and gap junction activities. Cx43 protein expression was also found to be downregulated in human tumor tissues as well as in several breast cancer cells when compared to their normal counterparts. Laird et al. [57] showed that reduced Cx43 expression can be used as an independent marker for the detection of breast tumors. Lack of Cx43 gap junctions was observed in ductal carcinomas* in situ*, infiltrating ductal carcinomas and infiltrating lobular carcinomas with no correlation with the level of estrogen and progesterone, the hormones that regulate Cx43 expression.

Cx26 was also found to be expressed at low levels in breast tumor tissues. The reason for the repression of Cx26 gene in breast tumors is clearly not known, though occurrence of methylation at the promoter region could contribute to the gene silencing [58, 59]. Support from this notion came from the work of Singal et al. [58], where they found that Cx26 is hypermethylated in MCF-7 breast cancer cells leading to gene silencing and reduced expression. Another study with tumor tissues from breast cancer patients reported that the Cx26 promoter was methylated in more than 50% of the tissues irrespective of the stage of cancer [59]. However there are some evidences that show connexins are highly expressed in breast carcinoma. Jamieson et al. [60] also showed that Cx26 expression was detected in 75% of breast ductal carcinoma* in situ*, a value which is much higher relative to normal breast tissues. Another study reported increased expression of Cx26 and Cx43 in lymph node metastases [61].

Another connexin, connexin46 (Cx46), has been implicated in early breast tumor growth (Table 2). Cx46 was found to be expressed as both mRNA and proteins, in MCF-7 cells and breast tumor tissues [62]. However the expression of Cx46 was not seen in normal human mammary epithelial cells (HMEC). Cx46 appeared to play a protective role against hypoxia induced death in breast cancer and retinoblastoma [62, 63]. Knockdown of Cx46 reduced MCF-7 viability under hypoxia and inhibited MCF-7 xenograft tumor growth in nude mice [62]. It was also reported that Cx46 and Cx43 display reciprocal relationship in expression which was regulated in proteasome mediated protein degradation pathway [63, 64].

The notion that connexins act as tumor suppressors was also supported by evidences where overexpression or reexpression of connexins was shown to reduce cell proliferation and tumor growth. Overexpression of Cx43 decreased proliferation of MCF-7 breast cancer cells in both 2D and 3D cultures [65]. Overexpression of Cx43 reduced MDA-MB-231 cell proliferation in 3D culture and retroviral delivery of Cx43 inhibited xenograft tumor growth* in vivo* without increasing membrane gap junctions [66].

## 6. Connexins in Breast Cancer Metastasis

**Figure 1:**
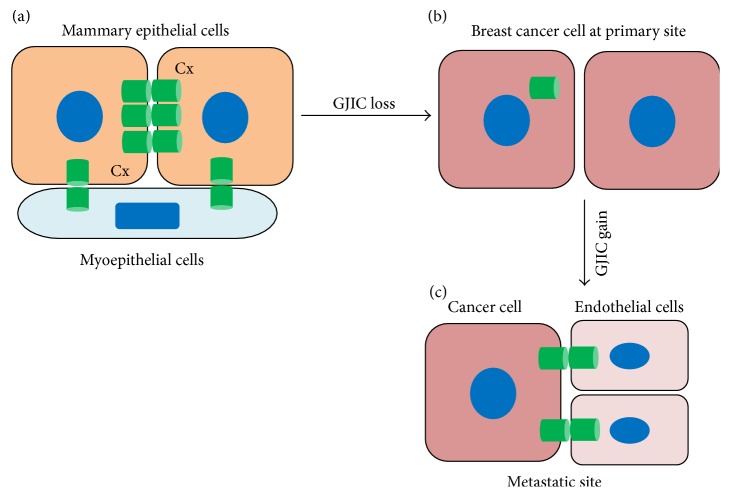
Gap junction connexins in the growth and progression of breast cancer. (a) In normal mammary gland, epithelial cells and myoepithelial cells maintain intercellular communication through gap junction connexins. (b) During breast cancer growth at primary site, loss of GJIC and low levels of connexins are observed. (c) As the cancer progresses tumor cells regain connexins expression and maintain GJIC with endothelial barrier that induce extravasation and adherence to the metastatic site.

The evidence presented above supports the idea that primary tumor progression is generally accompanied with reduced connexins expression and subsequent loss of gap junctional intercellular communication (Figure 1). However, connexins are generally deemed metastatic inducers. It is believed that loss of connexins gap junctions allows cells to physically detach from tumor microenvironment that increases cell's migratory ability. In later stages of cancer progression connexins are upregulated that aid tumor cells to invade, interact with endothelial cells, and extravasate to distant organs (Figure 1). Several studies have shown that connexins enhance a tumor cell's ability to metastasize through enhancing migration, invasion, and adhesion to tumor microenvironment. Overexpression of Cx43 in 4T1 cells increased the adhesion of these cells in pulmonary endothelium [67]. However, tumor cell adhesion was markedly decreased when a dominant negative Cx43 was overexpressed. There are several reports that showed increased expression of connexins in breast cancer metastatic tissues. Cx26 and Cx43 expression were detected in more than 50% of the invasive breast carcinomas, as compared to normal tissue samples [60]. In these tumor samples Cx43 were only detected in the cytoplasm and the presence of Cx43 gap junctions was not observed. Increased expressions of Cx26 and Cx43 were detected in lymph node metastases of breast cancer [68]. Primary tumors from these patients showed negative staining of these two connexins. Moreover, membranous staining of Cx26 and Cx43 was observed in the metastatic lymph node. Another study by the same group of researchers has shown that Cx32 has increased expression in lymph node metastasis of human ductal breast cancer [69]. 70% of primary tumors with lowly expressed Cx26, Cx32, and Cx43 developed lymph node metastases that expressed high levels of all three connexins [69]. This suggests tumors increased the expression of connexins at later stages of cancer progression.

Cx43 can also induce invasion and metastasis through the regulation of interaction between stromal cells and tumor cell. Stromal cell in pancreatic tumor microenvironment had been found to express Cx43 suggesting Cx43 may play tumor extrinsic roles in pancreatic cancer progression [70]. Cx43 and Cx26 were also reported to initiate brain metastatic lesion formation in association with the vasculature [71]. Knockdown of these two connexins by RNAi or the treatment with GJIC blocker carbenoxolone exhibited reduced brain colonization of tumor cells by inhibiting extravasation and blood vessel cooption. Metastatic breast cancer cells that are selected for their ability to colonize in the brain had increased expression of Cx43 suggesting that Cx43 is restricted in small population of breast cancer stem cells [72]. Connexins also enhance tumor cell adhesion to the metastatic organs. Enhancing GJIC, due to Cx43 overexpression, increased breast cancer cell adhesion to the lung [67]. Such adhesion was found to be inhibited when GJIC was impaired due to the presence of a dominant negative Cx43 [67] highlighting importance of connexins mediated GJIC activity in metastatic homing of breast cancer cells.

The role of Cx43 in all stages of breast tumorigenesis was investigated using transgenic mice. A study done by Plante et al. [73] showed that when Cx43 mutant mice were crossed with mice overexpressing oncogene ErbB2 the resultant mice displayed delayed onset of palpable tumors and extensive mammary gland hyperplasia. However these mice exhibited an increased lung metastatic burden. This data indicated that owing to its differential interaction with tumor microenvironment Cx43 can act as a tumor suppressor in early breast cancer growth and can also function as tumor enhancer in later stages of breast cancer progression.

## 7. Connexins in Breast Cancer Angiogenesis

Cx26 and Cx43 are also considered to be involved in the regulation of angiogenesis in breast cancer. Inhibition of gap junction activity by palmitoleic acid (PA) or siRNA-knockdown of Cx37, Cx40, or Cx43 diminished capillary networks of HUVEC* in vitro* angiogenesis assay [74]. Overexpression of a nonfunctional Cx26 variant resulted in the upregulation of an antiangiogenic molecule, thrombospondin-1 (TSP-1), in MDA-MB-435 cells [75] via gap junction independent mechanism. Similar results were obtained in a study where downregulation of Cx43, by a siRNA, reduced the expression of angiogenesis inhibitor TSP-1 and increased the expression of vascular endothelial growth factor (VEGF) leading to an aggressive cell phenotype of breast cancer Hs578t cells [76]. These findings were supported by another study where both Cx26 and Cx43 were overexpressed in MDA-MB-231 cells and conditioned media from 3D culture was probed with an angiogenesis antibody array [77]. Several angiogenesis-linked proteins such as IL-6 or MCP-1 (monocyte chemotactic protein-1) were upregulated by both Cx26 and Cx43 overexpression. Additionally, conditioned media from connexin overexpressing cells inhibited endothelial cells tubulogenesis and migration* in vitro*.* In vivo* Cx43 overexpressing MDA-MB-231 cells xenograft in nude mice displayed reduced tumor vasculature. Cx43 also reduced the expression of hypoxic-induced factor-1*α* (HIF-1*α*), a master transcription factor for angiogenesis, in astrocytes. Knockdown of Cx43 in 4T1 cells exhibited an increased VEGF and enhanced the proliferation of endothelial cells [78]. Cx43 overexpressed lung cancer cells B16F10, when subcutaneously transplanted in nude mice, resulted in inhibited tumor growth and angiogenesis. In all of the above study overexpression of connexins in tumor cells did not result in restoration of gap junction. Therefore connexins appear to inhibit angiogenesis and tumor growth via gap junction independent mechanism.

## 8. Connexin and Other Signaling Pathways

Independently of their junctional activity connexins are also reported to interact and modulate several signaling pathways implicated in breast cancer. Heterocellular interaction between SCg6 and SCp2, the epithelial and myoepithelial subclones of CID-9 mouse mammary cells, increased the association of Cx32, Cx43, and Cx30 leading to loss of nuclear localization and recruitment into the membrane *β*-catenin [42]. This recruitment of *β*-catenin into the membrane resulted in gap junction stabilization and induced gap junction mediated differentiation of mammary epithelial cells. Cx43 was also linked with other tight and adherent junctional proteins in breast cancer cells. In MCF-7 and MDA-MB-231 cells, overexpression of Cx43 reduced cell proliferation that was associated with reduced level of nuclear *β*-catenin though the total levels of *β*-catenin, *α*-catenin, and ZO-2 were not altered [79]. The GJIC independent roles of connexins have also been found in the regulation of apoptosis. Kanczuga-Koda et al. [80] found that Cx26 and Cx43 expression correlated with proapoptotic factor Bak but not with Bcl-2.

Cx43 pseudogene has also been identified and implicated in breast cancer [81]. Pseudogenes are generally deemed nonfunctional copies of DNA. However, Cx43 pseudogene (*ΨCx43*) is transcribed and expressed in MDA-MB-231, MDA-MB-435, and MCF-7 breast cancer cell lines but not in normal cells [81]. A study shows that protein corresponding ΨCx43 gene acts as a posttranscriptional regulator of* Cx43* in breast cancer cells [82]. The exogenous expression of this protein inhibits Cx43 translation by an unknown mechanism. However* ΨCx43* can bind more efficiently to the translational machinery than does* Cx43 and gene silencing of  ΨCx43* results in an increase of Cx43 RNA and proteins in breast cancer cells [82]. These results in increased cellular sensitivity to cytotoxic chemotherapy indicating Cx43 pseudogene have therapeutic potentials.

GJIC has also been implicated in the breast cancer metastasis through the exchange of miRNAs between cancer cells and bone marrow stromal cells. It has been shown that miRNA exchanged from bone marrow stroma to breast cancer cells via gap junctions resulted in cycling quiescence of the tumor cells and was also associated with lowered levels of CXCL12 [82]. Several miRNAs such as miR-127, miR-197, miR-222, and miR-223 had been identified, which target CXCL2, and found to be transported from bone marrow stroma to breast cancer cells causing decreased proliferation and arrest at G0 phase of cancer cell cycle [82]. Another miRNA, miR-206, was also discovered to be negatively correlated with Cx43 in metastatic axillary lymph node suggesting that miR-206 may inversely regulate Cx43 expression and function [83]. miR-206 which targets Cx43-3′UTR strongly decreased Cx43 expression in MCF-7 cells [83]. The association between miRNAs and Cx43 in the regulation of breast cancer was further supported by an evidence where another miRNA, miR-200a, was identified as a novel suppressor of connexin43 in breast cancer cells [84]. Decreased levels of miR-200a were found to be associated with elevated expression of Cx43 in the metastatic breast cancer tissues compared with the primary tumors [84].

## 9. Targeting Connexins in Breast Cancer

Due to the differential expression and functions of connexins at various stages of breast carcinogenesis several therapeutic strategies have been developed to modulate connexins or gap junctions expression in order to exert antitumor effects* in vitro* or* in vivo *(Table 1). Lack of connexin gap junctions in primary breast tumor has been utilized for therapeutic invention. As discussed before, overexpression or reexpression of connexins had been shown to exert antitumor activities in breast cancer cells. Several agents have also been developed to induce connexin expression or GJIC activities and tested for the potent antitumor efficacies (Table 3). These agents include quinolone organochlorine compounds, quinolone derivatives, and peptide mimetic. Organochlorine compound TCDD has been tested extensively in breast cancer cell lines and mouse models [85]. TCDD has been shown to inhibit gap junctional activity in MCF-7 cells which was associated with an increase in the phosphorylated Cx43 and PKC*α* [85]. TCDD also caused a decrease in GJIC in human mammary epithelial cells (HMEC) [85]. Similarly, a quinolone derivative compound PQ1 has been used extensively to target connexins in breast cancer. PQ1, which has a strong binding affinity for Cx43, has been shown to exert potent antitumor effects on breast cancer cell lines [86]. PQ1 treatment resulted in a decrease in GJIC and colony formation ability of T47D cells [86]. PQ1 treatment also reduced T47D xenograft tumor growth in nude mice [86]. Combination treatment of PQ1 with tamoxifen increased cytotoxic effects of PQ1 in T47D cells [86]. Interestingly, PQ1 treatment had no anticonnexin effects in normal mammary epithelial cell lines suggesting this compound has selective mode of action for cancer cell line. The effect of PQ1 treatment on tumorigenesis and metastasis was also evaluated in the MMTV-polyoma-middle-T genetically engineered mouse model [87]. Treatment with PQ1 significantly reduced tumor growth in three stages of development: pre-, early-, and late-tumor formation [87]. PQ1 treatment increased Cx43 expression during pre- and early-tumor formation further supporting the concept that this connexin has a tumor suppressor role in early-tumor growth [87]. Though these quinolone derivatives showed excellent antitumor effects on breast cancer their mode of action is not selective. PQ1 also influences other signaling pathways such as Akt and MAPK pathways and therefore has the ability to induce off-target effects in SW480 human colorectal cancer cells [88].

The phenomenon “bystander effect” also had been therapeutically utilized where restoration or augmentation of gap junction activities increased intracellular signaling among neighboring cells and provided better delivery of drugs to induce cell deaths. For example, all-*trans*-retinoic acid (ATRA) had been shown to enhance GJIC by increasing Cx43 expression. When MCF-7 cells were treated with ATRA, in combination with a VEGFP-TK/CD gene suicide system, the cells were found to undergo increased apoptotic death by strengthening bystander effects [89]. PQ1 treatment also increased bystander effects and synergized with the cytotoxic effects of cisplatin in breast cancer cells. Combination treatment of PQ1 and cisplatin increased the expression of Cx26, Cx32, and Cx43 and GJIC in T47D cells which was accompanied with reduced cell proliferation as compared to either single treatment [86]. This combination therapy increased the expression of proapoptotic molecules such as caspases 3, 8, and 9 and decreased the prosurvival Bcl2 protein [86].

Peptide-based targeting of connexins has also shown potent antitumor activities. A unique 25-amino acid length peptide drug (ACT1), which mimics a cytoplasmic regulatory domain of Cx43, has been tested in breast cancer for its potent antitumoral activity [90]. This peptide drug increases gap junction aggregation by redirecting uncoupled Cx43 hemichannels into the membrane thereby increasing gap junction function without altering expression level of Cx43 [90]. Targeting Cx43 with ACT1 peptide reduced MCF-7 and MDA-MB-231 cell proliferation* in vitro* in a dose dependent manner [90]. However this peptide had no antiproliferative effects on mammary epithelial MCF10A cells. ACT1 had also been shown to enhance intercellular coupling between cells and this effect has been utilized to test whether it can enhance the efficacy of cytotoxic drugs [90].

## 10. Conclusion

Connexins play multiple roles in normal mammary gland development and homeostasis, as well as in breast cancer progression (Table 2). Cx26 is expressed in luminal cells and Cx43 is expressed in myoepithelial cells of normal human mammary gland. The expression of connexins is important for the maintenance of intercellular communication among cells of mammary gland during lactation and pregnancy. During the growth of breast tumor Cx26 and Cx43 are expressed at low levels and tumor growth is facilitated by lack of GJIC. Reexpression of connexins to breast cancer cells reduces proliferation* in vitro* and inhibits tumor growth* in vitro*. However, one connexin, Cx46, has been reported to have higher expression in breast cancer cell lines and act to protect tumor cells from hypoxia induced death. During cancer progression lack of connexin's connections helps tumor cells to physically detach from microenvironment and migrate. Several studies have shown that in later stages of cancer progression connexins are upregulated that aid tumor cells to interact with endothelium, extravasate, and adhere to distant organs. Therefore depending on the stages of breast cancer connexins can act as tumor suppressor or tumor inducer. There are several methods or agents that have been developed to increase connexin expression or GJIC to revert tumor cells characteristics. However the thought that connexins can be used for ultimate therapeutic target is far from convincing at the current time. Further studies are needed to investigate the functions of connexins hemichannels in carcinogenesis and tumor progression. Though connexins are upregulated during metastasis no drug to date has been developed or tested to target connexin in metastasis. Studies along these lines with more functional, gap junction dependent or independent, characterization of connexins and the investigation of cross talk with other signaling pathways in breast tumor oncogenesis would be useful in fully elucidating the therapeutic potential of targeting connexins in breast cancer.

## Figures and Tables

**Table 1:** Family of connexin genes. Adopted from Beyer and Berthoud [7].

Human connexin		Mouse connexin	
CX	GJ	Cx	Gj
CX43	GJA1	Cx43	Gja1
CX46	GJA3	Cx46	Gja3
CX37	GJA4	Cx37	Gja4
CX40	GJA5	Cx40	Gja5
—	—	Cx33	Gja6
CX50	GJA8	Cx50	Gja8
CX59	GJA9 (GJA10)	—	—
CX62	GJA10	Cx57	Gja10
CX32	GJB1	Cx32	Gjb1
CX26	GJB2	Cx26	Gjb2
CX31	GJB3	Cx31	Gjb3
CX30.3	GJB4	Cx30.3	Gjb4
CX31.1	GJB5	Cx31.1	Gjb5
CX30	GJB6	Cx30	Gjb6
CX25	GJB7	—	—
CX45	GJC1 (GJA7)	Cx45	Gjc1
CX47	GJC2 (GJA12)	Cx47	Gjc2
CX30.2/CX31.3	GJC3 (GJE1)	Cx29	Gjc3
CX36	GJD2 (GJA9)	Cx36	Gjd2
CX31.9	GJD3 (GJC1)	Cx30.2	Gjd3
CX40.1	GJD4	Cx39	Gjd4
CX23	GJE1	Cx23	Gje1

**Table 2:** The connexins and their expression, localization, and function in normal breast and breast tumor.

Connexin	Normal breast		Breast tumor	
	Expression	Function	Expression	Function
Cx26	Luminal epithelial cells	Luminal cell proliferation Production of milk	Downregulated in primary tumor Upregulated in metastatic tissue	
Cx43	Myoepithelial cells Decreased during mid-pregnancy and lactation	Proper production and ejection of milk	Downregulated in primary tumor Upregulated in metastatic tissue	Increases invasion, adhesion of tumor cells
Cx30	Mouse luminal epithelial cells Peak expression at the onset of lactation	Lactation Compensates for impaired Cx32		
Cx32	Mouse luminal epithelial cells Increased expression at parturition	Production of milk Compensates for impaired Cx30 function	Increased expression at metastatic lymph node	
Cx46	Expression not reported in mouse or human normal breast		Expressed in primary tumor	May protect tumor cells from hypoxia induced death

**Table 3:** Drugs, their mode of action, and reported effects on GJIC and breast tumor.

Drug	Mode of action	Anti-breast tumor effect
Organochlorine compound TCDD	Nonspecific, increases phosphorylated Cx43 and GJIC	Decreases GJIC
PQ1 quinolone derivative	Nonspecific, increases Cx43 expression and increases GJIC	Decreases colony formation ability and xenograft tumor growth of T47D cells
PQ1 + cisplatin	Combination treatment, bystander effect	Reduces T47D cell proliferation synergizing with cytotoxic effects of cisplatin
Peptide ACT1	Increases gap junction aggregation by redirecting uncoupled Cx43 hemichannels into the membrane	Reduces MCF-7 and MDA-MB-231 cell proliferation *in vitro*
ACT1 + tamoxifen	Combination treatment, bystander effect	Augments tamoxifen cytotoxic effects on MCF-7 cells
All-*trans*-retinoic acid (ATRA) + VEGFP-TK/CD gene suicide system	ATRA increases GJIC and mediates bystander effects to induce cell killing by gene suicide system	Increases apoptotic MCF-7 cell death
Anti-Cx46	Knocks down Cx46	Reduces MCF-7 viability under hypoxia and inhibits xenograft tumor growth
